# Systemic inflammatory markers in relation to cognitive function and measures of brain atrophy: a Mendelian randomization study

**DOI:** 10.1007/s11357-022-00602-7

**Published:** 2022-06-11

**Authors:** Jiao Luo, Saskia le Cessie, Gerard Jan Blauw, Claudio Franceschi, Raymond Noordam, Diana van Heemst

**Affiliations:** 1 Department of Clinical Epidemiology, Leiden University Medical Center, Leiden, The Netherlands; 2 Department of Internal Medicine, Section of Gerontology and Geriatrics, Leiden University Medical Center, PO Box 9600, 2300RC Leiden, The Netherlands; 3 Department of Biomedical Data Sciences, Leiden University Medical Center, Leiden, The Netherlands; 4 Department of Experimental, Diagnostic and Specialty Medicine (DIMES), University of Bologna, Bologna, Italy

**Keywords:** Inflammation, Cytokines, Cognitive function, Hippocampal volume, Cerebral cortex

## Abstract

Observational studies have implied associations between multiple cytokines and cognitive decline, anti-inflammatory drugs however did not yield any protective effects on cognitive decline. We aimed to assess the associations of systemic inflammation, as measured by multiple cytokine and growth factor, with cognitive performance and brain atrophy using two-sample Mendelian randomization (MR). Independent genetic instruments (*p* < 5e − 8 and *p* < 5e − 6) for 41 systemic inflammatory markers were retrieved from a genome-wide association study conducted in 8293 Finnish participants. Summary statistics for gene-outcome associations were obtained for cognitive performance (*N* = 257,841) and for brain atrophy measures of cerebral cortical surface area and thickness (*N* = 51,665) and hippocampal volume (*N* = 33,536). To rule out the heterogeneity in the cognitive performance, we additionally included three domains: the fluid intelligence score (*N* = 108,818), prospective memory result (*N* = 111,099), and reaction time (*N* = 330,069). Main results were computed by inverse-variance weighting; sensitivity analyses taking pleiotropy and invalid instruments into account were performed by using weighted-median estimator, MR-Egger, and MR PRESSO. After correcting for multiple testing using false discovery rate, only genetically predicted (with *p* < 5e − 6 threshold) per-SD (standard deviation) higher IL-8 was associated with − 0.103 (− 0.155, − 0.051, *p*_adjusted_ = 0.004) mm^3^ smaller hippocampal volume and higher intelligence fluid score [*β*: 0.103 SD (95% CI: 0.042, 0.165), *p*_adjusted_ = 0.041]. Sensitivity analyses generally showed similar results, and no pleiotropic effect, heterogeneity, or possible reverse causation was detected. Our results suggested a possible causal association of high IL-8 levels with better cognitive performance but smaller hippocampal volume among the general healthy population, highlighting the complex role of inflammation in dementia-related phenotypes. Further research is needed to elucidate mechanisms underlying these associations.

**Supplementary Information:**

The online version contains supplementary material available at 10.1007/s11357-022-00602-7.

## Introduction

Dementia has become a major global health concern due to increased longevity and increased number of people aged 60 years and older. The population living with dementia is estimated to triple to approximately 150 million by 2050 from now [1, 2]. Changes in the brain start to occur several years before the first manifestation of clinical symptoms and diagnosis of dementia [3]. This indicates a large time window to delay or even prevent the onset of clinically significant cognitive deficits. In the absence of any effective pharmacologic preventive and/or curative treatment to date [4], early markers would therefore provide insight into the pathogenesis and targets for potential preventive strategies.

Systemic inflammation has been hypothesized as a risk factor in cognitive decline and dementia [5–8]. Epidemiological studies have identified associations of elevated systemic inflammation levels and worse cognition in cross-sectional studies, and a steeper cognitive decline in prospective studies [9, 10]. Moreover, anti-inflammatory therapy, particularly nonsteroidal anti-inflammatory drugs (NSAIDs), has been associated with a lower risk of cognitive decline in a meta-analysis of observational cohort studies [11]. However, in a recent systematic review of randomized clinical trials (RCTs), aspirin or other NSAIDs did not lower the risk of dementia [12]. Several potential explanations could have been contributed to these inconsistencies. Most importantly, associations from observational studies are prone to reverse causality and residual confounding. In addition, NSAIDs might be beneficial only when used in the very early stages of cognitive deficits [13]. Intervention in RCTs that have been carried out might be too late to modify the progression of cognitive decline. Therefore, it remains to be elucidated whether systemic inflammation is causally related to cognitive performance.

As such, Mendelian randomization (MR) is an alternative approach using genetic instruments that are randomly allocated at conception as a proxy of exposure to infer the causality of life-long exposure on disease [14]. Three recent MR studies on inflammatory markers and Alzheimer’s disease (AD), the most common type of dementia, have yielded inconsistent results [15–17]. Despite being done in large populations, these studies could still suffer from limited power caused by a limited number of cases. Continuous traits are generally acknowledged to have more statistical power, and therefore, cognitive function and measures of brain atrophy, which are hallmark characteristics of AD and other forms of dementia [18, 19], might be better suitable phenotypes to dissect the potential causation between inflammation and dementia.

Given that inflammation is a complex process regulated through an integrated network of pro- and anti-inflammatory immune cells and cytokines, investigating multiple inflammatory markers simultaneously in the same study population could provide more insights into the role of inflammation in dementia. Therefore, in the present study, we leveraged a two-sample MR to assess causality in relation to a comprehensive amount of 41 genetically predicted circulating levels of systemic inflammatory markers with general cognitive performance (with three additional domains of the fluid intelligence score, prospective memory result, and reaction time) and measures of brain atrophy (cerebral cortical surface area and thickness and hippocampal volume).

## Method

### Study design

**Fig. 1:**
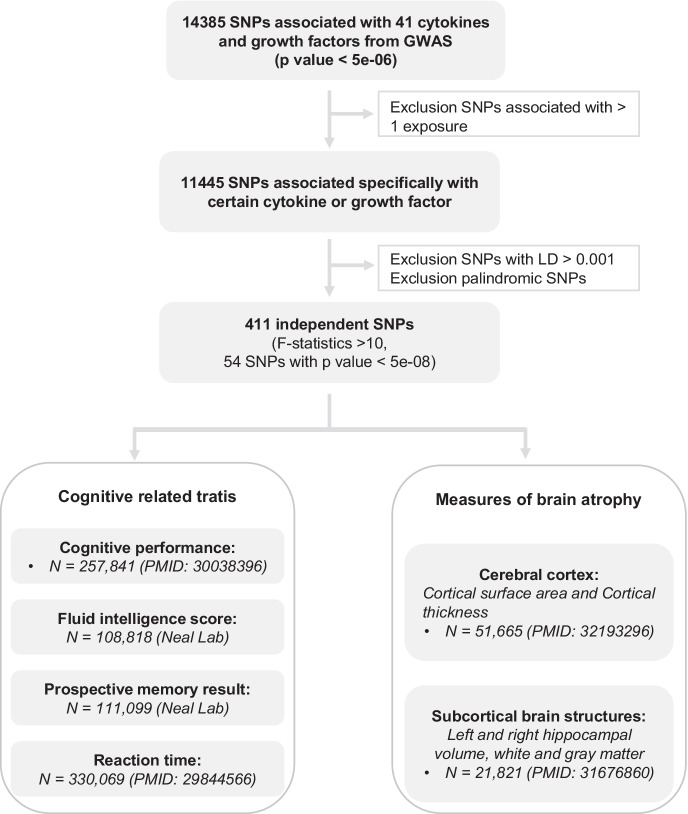
Schematic overview of the study design

We conducted a two-sample MR study, and the overview of the study design is presented in Fig. 1. MR builds on three principal assumptions: the instrumental variables should firstly be associated with the exposure; secondly not be associated with confounding factors in the relation between exposure and outcome; thirdly affect the outcome exclusively via the exposure, but not via other pathways. Data involved in the present study are publicly available summary statistics from genome-wide association study (GWAS). Specific ethical approval and informed consent were obtained in the original studies.

### Selection of instrumental variables

Genetic instrumental variables associated with 41 circulating cytokines and growth factors were obtained from a recent GWAS, which was conducted in 8293 randomly chosen participants from five geographical areas of Finland aged between 25 and 74 years, including The Cardiovascular Risk in Young Finns Study (YFS), FINRISK 1997, and FINRISK 2002 [20] (data were downloaded from http://computationalmedicine.fi/data#Cytokine_GWAS). All gene-exposure associations were reported as regression coefficients (*β*) in SD (standard deviation) -scaled units, adjusted for age, sex, body mass index, and the first 10 genetic principal components.

**Fig. 2:**
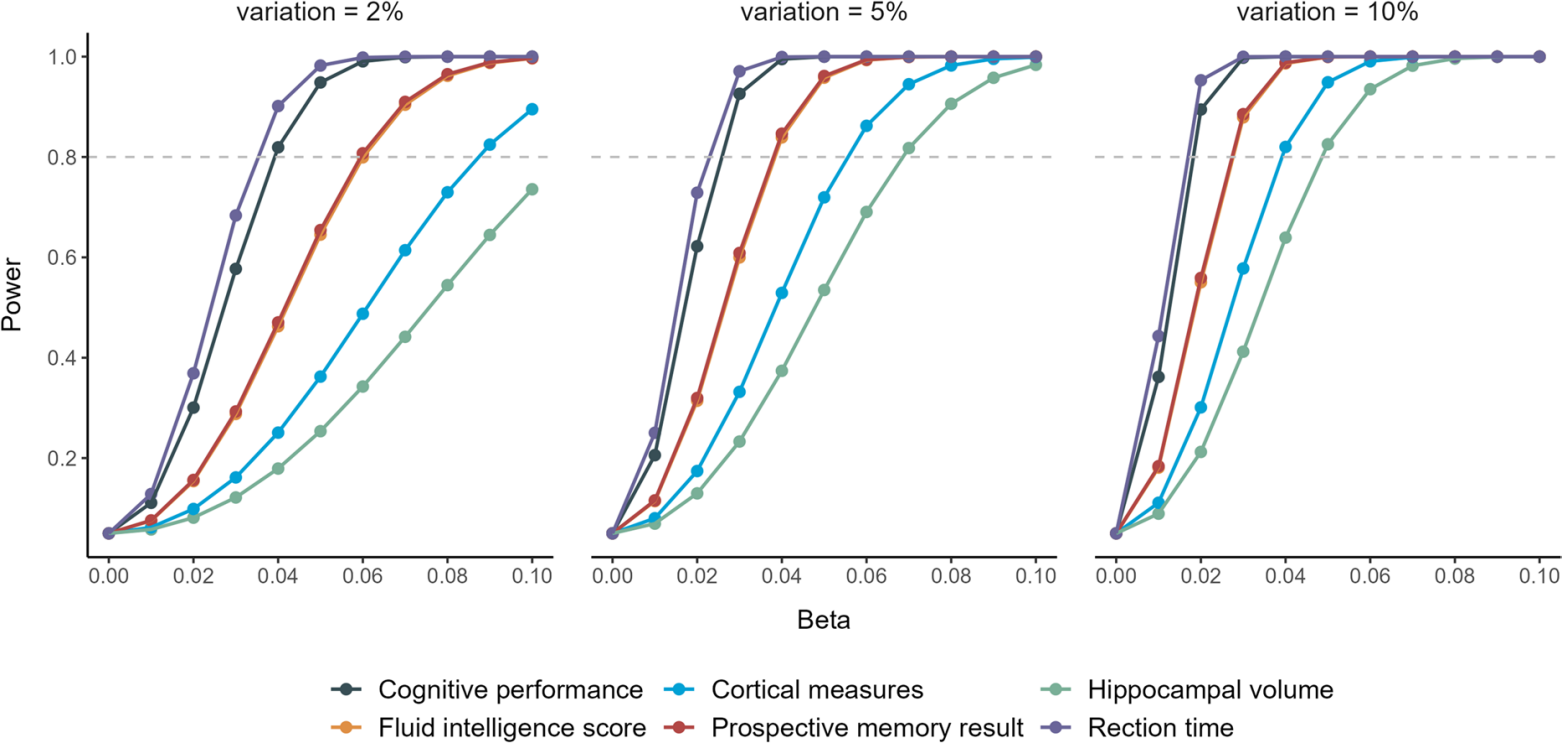
Statistical power for each outcome in MR analyses with different variations explained by the genetic instrumental variables

To avoid pleiotropic bias, we first excluded SNPs that were associated with more than one cytokine and/or growth factor. Linkage disequilibrium (LD) between all SNPs for the same exposure was assessed in the European 1000 Genome Project reference panel. When LD presented (LD > 0.001), the variant with the smallest *P*-value was retained. Since many markers had no or very few (<3) significant single nucleotide polymorphisms (SNPs) at the genome-wide significant level (*p* < 5e − 8), we also adopted a more relaxed significance threshold (*p* < 5e-6) for instrumental variables selection. To minimize weak instrumental bias, *F* statistics was calculated to assess the strength of each instrument, and a value of above 10 is considered sufficient. The proportion of total variation (*R*^2^) explained by the individual genetic instrument was calculated using the formula $R^2={(\beta\times\sqrt{2\times MAF\left(1-MAF\right)})}^2$, where *β* is the effect of the genetic variant on the respective cytokine levels and MAF is the minor allele frequency [21]. When MAF is not presented in the original dataset, we extracted the effect allele frequency from PhenoScanner GWAS database Version 2. *R*^2^ for each exposure was calculated in an additive model of all included SNPs assuming no interaction between the individual SNPs. In addition, we calculated statistical power based on the online tools for continuous outcomes (https://github.com/kn3in/mRnd) (9), where the alpha level was set to 0.05. When the variance explained by the genetic variants was at the minimum of 2%, we had adequate power of 0.8 to detect about 0.04, 0.088, 0.11, 0.06, 0.06, and 0.035 unit difference in cognitive performance (in SD), cortical measures (surface area in mm^2^ and thickness in mm), hippocampal volume (in mm^3^), fluid intelligence score (SD), prospective memory results (SD), and reaction time (SD), respectively (Fig. 2).

### Outcome data sources

#### Cognitive performance

Summary statistics for the genetic associations with general cognitive performance were extracted from a recent GWAS (*N* = 257,841) with European-descent individuals, using a sample-size-weighted meta-analysis to combine data from Cognitive Genomics Consortium (COGENT, *n* = 35,295) and the UK Biobank (*n* = 222,543) [22]. All individuals were aged between 16 and 102 years without stroke or prevalent dementia. In COGENT, cognitive performance was defined as the score on the first unrotated component of the performance of at least three different neuropsychological tests within each included study. In the UK Biobank, verbal numerical reasoning (VNR) was assessed by 13 multiple-choice questions, and the VNR score was determined as the number of questions answered correctly with a time limit of 2 min, designed as a measure of a fluid intelligence test. This test has been demonstrated to have adequate reliability and validity [22, 23]. Since the majority of the associations, as reflected in the number of participants, are derived from the UKB, the final effects are more representative for the executive function measured by the fluid intelligence test. Genetic association estimates are presented in SD units.

Due to the heterogenous definition of cognitive performance in the above GWAS, we additionally included three phenotypes to represent different domains, namely, the fluid intelligence score (*N* = 108,818) and prospective memory result (*N* = 111,099) from the UK Biobank conducted by the Neal Lab (https://www.neallab.org/), and reaction time (*N* = 330,069) from the COGENT. Fluid intelligence score is a simple unweighted sum of the number of correct answers given to the 13 fluid intelligence questions. Therefore, we included this phenotype as a holistic measurement of multiple domains of “fluid intelligence”. For prospective memory, participants were allowed up to 2 attempts to correctly recall the color/shape that was shown to them earlier in the touchscreen section, and it condenses the results into 3 groups: instruction not recalled, either skipped or incorrect; correct recall on first attempt; and correct recall on the second attempt. Reaction time is based on 12 rounds of the card-game “Snap”. The participant is shown two cards at a time; if both cards are the same, they press a button-box that is on the table in front of them as quickly as possible. For each of the 12 rounds, the following data were collected: the pictures shown on the cards, the number of times the participant clicked the “snap” button, and the time it took to first click the “snap” button.

#### Cerebral cortical surface area and thickness

Genetic associations with cerebral cortical surface area (mm^2^) and thickness (mm) were obtained from a genome-wide association meta-analysis of brain T1-weighted magnetic resonance imaging (MRI) data from 51,665 predominantly healthy individuals of European ancestry aged between 3.3 and 91.4 years across 60 cohorts by Enhancing Neuroimaging Genetics through Meta-analysis (ENIGMA) consortium [24]. Almost all participants are healthy, except for less than 1% with psychiatric disorders from included case–control studies out of the 60 cohorts. The cortical surface area was measured at the grey-white matter boundary, and thickness was measured as the average distance between the white matter and pial surfaces. The total surface area and average thickness were computed for each participant separately. The genetic associations were calculated using an additive model within each cohort, adjusted for age, age squared, sex, sex-by-age interactions and age squared, the first four multidimensional scaling components, and diagnostic status (when the cohort followed a case–control design) and dummy variables for scanner when applicable.

#### Hippocampal volume

For gene-hippocampal volume associations, a GWAS meta-analysis from high-resolution brain MRI scans in 33,536 healthy individuals aged between 11 and 98 years at 65 sites between the ENIGMA and the CHARGE consortia was used; mean bilateral hippocampal volume (mm^3^) was defined as the average of left and right [25]. Genetic associations in the study were assessed within each site, adjusted for age, age squared, sex, intracranial volume, four multidimensional scaling components, and diagnostic status when applicable; site effects were also adjusted for studies with data collected from multiple centers or scanners; mixed-effects models were additionally used to account for familial relationship with family data. Since only the *z*-statistic and *p*-value for each SNP were provided in the dataset, we calculated the corresponding estimate of the standardized regression coefficient for an outcome on an genetic variant ($\widehat{\beta }$) based on the equation $\widehat{\beta }$ = *z* statistic × se in the previous study, and se is computed by $\text{se = 1/}\sqrt{2\times\mathrm{MAF}\left(1\,-\,\mathrm{MAF}\right)(\mathrm N+\mathrm z\;\mathrm{statistic}^2)}$, where MAF is the minor allele frequency and *N* is the sample size [26].

### Statistical analysis

We harmonized exposure and outcome GWAS summary statistics by making alignment of the summary statistics to the forward strand if the forward strand was known or could be inferred. Palindromic SNPs, that could not to be inferred to the forward strand and can introduce ambiguity into the identification of the effect allele in the exposure and outcome GWAS, were removed [27].

For the main analysis, inverse-variance weighted (IVW) regression analysis was used, which assumes no directional pleiotropic effects of individual instrumental variable [28]. This estimate combines the SNP-specific Wald ratios (gene-outcome association divided by gene-exposure association) by using a meta-analysis weighted by the inverse of the variance of the Wald estimates. Results are expressed as per unit change in regression coefficient (95% confidence interval) on the outcome of per standard deviation (SD) change in inflammatory markers. We also performed additional sensitivity analyses that take pleiotropy into account, including MR-Egger, weighted-median estimator, and MR PRESSO (Pleiotropy Residual Sum and Outlier) [29–31]. In particular, the MR-Egger regression intercept estimates the average pleiotropic effect across the genetic variants if the MR assumption and the InSIDE (INstrument Strength Independent of Direct Effect) assumption hold [29]. An intercept that differs from zero indicates the presence of directional pleiotropy. A weighted-median estimator analysis can provide a valid estimate if at least half of the instrumental variables are valid [30]. MR-PRESSO was applied when there were sufficient number of genetic variants to detect and correct for horizontal pleiotropy through removing outliers with the assumption of more than 50% valid instruments and balanced pleiotropy and InSIDE [31]. We used Cochran’s *Q* test statistic to examine the between-SNP heterogeneity; singe-SNP analysis was used to perform MR on each SNP individually, and leave-one-out analysis was performed to assess if one particular variant could potentially have driven the association.

### Reverse analysis

In order to examine the presence of possible reverse causation, we additionally tested the associations between genetically influenced cognitive performance and measures of brain atrophy with any of the 41 inflammatory markers. To this end, we extracted independent genetic variants at genome-wide significant level for cognitive performance, cerebral cortical surface area and thickness, and hippocampal volume, from the same GWAS when these are used as outcomes in the main analyses. We excluded SNPs that were both associated with cerebral cortical surface area and thickness to maximally eliminate pleiotropic effect.

All the analyses were undertaken using R (v3.6.3) statistical software (The R Foundation for Statistical Computing, Vienna, Austria). MR analyses were performed using the R package “TwoSample MR” and “MR PRESSO”. We used the false-discovery rate (FDR)-based multiple comparison with using Benjamini–Hochberg method, to correct for multiple testing. For cognitive performance and its domains, the adjustments were performed within each trait to avoid excessive stringency, whereas for cortical measure, the adjustment was performed for surface area and thickness simultaneously due to the same MRI measurement for these two traits. An adjusted *p*-value of 0.05 was considered as statistically significant.

## Results

### MR results

At the genome wide significance threshold (*p* < 5e − 08) for instrument selection, 9 out of 41 inflammatory markers with no less than 3 SNPs. *F*-statistics were between 29.9 and 789.1, and the variation explained by the genetic variants for each marker ranged from 1.7% for VEGF to 19.8% for MIP1b, as shown in Table 1*.* No significant associations between inflammatory markers and any of the outcome measures were observed after stringent correction for multiple testing.

**Table 1:** Summary information of genetic instrumental variables for each systemic inflammatory marker

Exposure	Full name	*p* < 5e − 08			*p* < 5e-06		
		**No. of SNPs**	**Variation (%)**	***F*-statistics (range)**	**No. of SNPs**	**Variation (%)**	***F*-statistics (range)**
bNGF	Nerve growth factor beta	1	1.1	36.5	8	5.3	20.8–36.5
CTACK	Cutaneous T cell-attracting chemokine	3	6.3	29.9–142.7	11	12.7	21.5–142.7
Eotaxin	-	3	2.3	32.5–95.2	16	6.8	20.8–95.2
FGFBasic	Basic fibroblast growth factor	0	-	-	6	2	20.8–24
GCSF	Granulocyte colony-stimulating factor	0	-	-	9	6.3	20.4–25.2
GROa	Growth-related oncogene alpha	2	6.3	41.8–184.4	10	14.4	20.7–184.4
HGF	Interleukin 10	2	1.5	40.8–57.3	9	4.4	20.7–57.3
IFNg	Interleukin 12p70	0	-	-	9	2.5	21.9–23.9
IL10	Hepatocyte growth factor	0	-	-	6	2	21.3–25.3
IL12p70	Interferon gamma	0	-	-	9	3.4	20.8–26.5
IL13	Interleukin 13	0	-	-	10	8.4	21.1–25.4
IL16	Interleukin 16	2	8.2	31.1–132	10	16.7	20.9–132
IL17	Interleukin 17	1	0.6	39	9	3.3	20.4–39
IL18	Interleukin 18	4	6.8	31.8–96.2	19	21.5	20.6–96.2
IL1b	Interleukin 1 beta	0	-	-	6	3.9	13.6–31.6
IL1ra	Interleukin 1 receptor antagonist	0	-	-	7	4.9	21.2–23.6
IL2	Interleukin 2	0	-	-	10	7	20.9–23.5
IL2ra	Interleukin 2 receptor alpha subunit	1	9.7	167.6–167.6	9	16.9	21.3–167.6
IL4	Interleukin 4	0	-	-	11	5.2	21.1–26.6
IL5	Interleukin5	0	-	-	5	3.8	22.1–24.9
IL6	Interleukin 6	0	-	-	7	2.7	21.6–23.3
IL7	Interleukin 7	0	-	-	12	12.3	20.6–26.4
IL8	Interleukin 8	0	-	-	3	4.8	22.1–24.6
IL9	Interleukin9	0	-	-	7	5.3	21.2–26.5
IP10	Interferon gamma-induced protein 10	2	1.9	31.1–32	12	10	21–32
MCP1	Monocyte chemoattractant protein-1	3	1.9	30.3–86.4	13	6	20.9–86.4
MCP3	Macrophage colony-stimulating factor	0	-	-	4	8.3	22–25.7
MCSF	Macrophage migration inhibitory Factor	1	1.6	31.6	8	13.3	21.3–31.6
MIF	Monokine induced by interferon-gamma	1	1.1	39–39	8	8.1	21.2–39
MIG	Interleukin 10	1	1	42.4	17	17.8	19–42.4
MIP1a	Macrophage inflammatory protein 1 alpha	0	-	-	9	6.4	21–22.8
MIP1b	Macrophage inflammatory protein-1 beta	7	19.8	58.2–789.1	22	26.2	20.6–789.1
PDGFbb	Platelet-derived growth factor BB	4	3.3	30.8–103.3	14	6.4	21–103.3
RANTES	Regulated on activation, normal T cell expressed and secreted	1	0.4	30	11	7.6	20.9–30
SCF	Stem cell factor	1	0.4	31.8	9	3.9	20.8–31.8
SCGFb	Stem cell growth factor beta	3	6.5	43.3–99.4	14	13.7	20.7–99.4
SDF-1α	Stromal cell–derived factor 1 alpha	0	-	-	9	3.6	11.2–29.2
TNFa	Tumor necrosis factor alpha	0	-	-	5	6.8	22.1–24.9
TNFb	Tumor necrosis factor beta	1	2	50.9	5	8.5	21.9–50.9
TRAIL	TNF-related apoptosis inducing ligand	7	18.5	99.4–370	19	24.4	19.9–370
VEGF	Vascular endothelial growth factor	3	1.7	33.4–42.9	14	6.5	20.8–42.9

The proportion of total variance (*R*^2^) explained by the individual genetic instrument was calculated using the formula $R^2={(\beta\times\sqrt{2\times MAF\left(1-MAF\right)})}^2$, where *β* is the effect of the genetic variant on the respective cytokine levels and MAF is the minor allele frequency

At a relaxed significance threshold (*p* < 5e − 06) for instruments selection for the other 32 markers, *F*-statistics of individual variants ranged from 11.2 to 789. Instruments for each marker explained the proportional variance from 2.0% for FGFBasic to 26.2% for MIP1b. The results are generally similar comparing to those obtained at *p* < 5e − 08 for instruments selection of the inflammatory markers when available, with however narrower confidence intervals (Supplementary Tables 1 and 2). In the primary analysis using IVW, genetically predicted one-SD higher IL-8 was associated with − 0.103 (− 0.155, − 0.051, *p*_adjusted_ = 0.004) mm^3^ smaller hippocampal volume. For different cognitive performance domains, higher IL-8 was however associated with higher intelligence fluid score [*β*: 0.103 SD (95% CI: 0.042, 0.165), *p*_adjusted_ = 0.041], as shown in Table 2. Estimations from the weighted-median method were generally consistent with those from IVW, and betas (95%CIs) were − 0.099 (− 0.169, − 0.282, *p*_adjusted_ = 0.23) and 0.10 (0.018, 0.182, *p*_adjusted_ = 0.4), respectively. No pleiotropic effect was detected via the MR-Egger intercept (both *p* value > 0.5). No between-SNP heterogeneity observed with the Cochran’s *Q* test statistic. Single-SNP analysis and leave-one-out analysis indicated that these associations were unlikely driven by a certain extreme variant (data not shown).

**Table 2:** Associations of systemic inflammation markers with measures of cognitive performance and measures of brain atrophy

Exposure	Outcome	Method^*^	No. of SNPs	Beta (95% CI)	*p*_adjusted_	Heterogeneity *Q* value (*p* value)	MR-Egger intercept (*p* value)
IL-8	Hippocampal volume (mm^3^)	IVW	3	− 0.103 (− 0.155, − 0.051)	0.004	1.1 (0.6)	
		Weighted-median	3	− 0.099 (− 0.169, − 0.282)	0.22		
		MR-Egger	3	− 0.073 (− 0.154, 0.008)	0.92	0.2 (0.7)	− 0.008 (0.5)
IL-8	Fluid intelligence score (SD)	IVW	3	0.103 (0.042, 0.165)	0.041	1.6 (0.6)	
		Weighted-median	3	0.102 (0.015, 0.186)	0.45		
		MR-Egger	3	0.103 (− 0.001, 0.208)	0.86	1.6 (0.3)	− 0.000 (1.0)

Genetic variants selection was based on *p* < 5e − 06. MR PRESSO (MR Pleiotropy RESidual Sum and Outlier) was not available due to limited number of SNPs. *p*_adjusted_ represents the FDR-based adjusted *p*-value

*IVW* inverse variance weighted

### Reverse analyses

In total, respectively 182, 121, 6, and 4 SNPs for cognitive performance, cerebral cortical surface area and thickness, and hippocampal volume that also presented in the inflammatory marker GWAS were identified. Upon correcting for multiple testing using FDR, no significant association was detected using IVW method (Supplementary Table 3). Results from sensitivity MR methods did not differ substantially compared to the IVW analyses.

## Discussion

In this two-sample MR analysis, we used genetic instruments for 41 systemic inflammatory markers to assess their potential causal associations with cognitive performance including its three domains (fluid intelligence score, prospective memory result, and reaction time), and measures of brain atrophy. Genetically predicted higher levels of IL-8 were associated with smaller hippocampal volume, but with better fluid intelligence score.

Systemic inflammation may result in neuronal consequences through several different pathways: (1) by interacting with blood–brain barrier function through receptor binding of inflammatory markers or through secretion of immune-active substances, (2) by neural afferent pathways bypassing the blood–brain barrier such as via the cranial nerve, and (3) through diffusion from blood through the perivascular spaces via the circumventricular organs which lack an endothelial blood–brain barrier [32–34]. IL-8 is a chemokine produced by several cell types and functions as a chemoattractant that recruits different types of immune cells to sites of inflammation by activating predominantly neutrophils, and it can act as a potent angiogenic factor. Higher IL-8 levels, either in circulation or cerebrospinal fluid, have been associated with poor cognitive performance [35, 36]. However, IL-8 measured in AD patients was found to be either elevated [5, 37], decreased [38], or unchanged [39]. Similarly, we also found conflicting results of IL-8, as it associated with both smaller hippocampal volume and better fluid intelligence score upon correction for multiple testing. However, interestingly, in consistent with these finding, genetically determined higher levels of IL-8 levels were also associated with better general cognitive performance [*β*: 0.026 SD (0.007, 0.045), *p*_original_ = 0.008] although before multiple testing correction only, and the same direction of effect on prospective memory score [*β*: − 0.016 SD (95% CI: − 0.045, 0.012)], *p*_adjusted_ = 0.8], although insignificant.

This inconsistency may be explained by several hypotheses. Foremost is the different domains of cognitive performance. A previous study specifically found that increased IL-8 was associated with worse cognitive performance in the memory and speed domains and in motor function [36]. While hippocampal volume is associated with amnestic, but not non-amnestic domain cognitive performance [40, 41], fluid intelligence in the present study was more of a reflection of executive function. Alternatively, the higher pro-inflammatory status induced by increased IL-8 might be counter-balanced by the production of higher levels of anti-inflammatory components, which may differentially affect cognition and hippocampal volume. As an example, while cortisol, which has potent anti-inflammatory effect and is often elevated in response to inflammation, may enhance alertness and memory, long-term exposure to cortisol may severely damage the hippocampus [42]. In addition, inflammation may cast a dual role in the pathogenesis of dementia-related conditions particularly in AD [43, 44]. Briefly, in the healthy state or preclinical stage of neurological diseases, a modest inflammatory response would be beneficial for the clearance of waste products, whereas in advanced stages, overreacted excessive inflammatory response that exceeds the capacity of self-repair would exacerbate the (neuro)inflammation. A fine example is that lower levels of both C-reactive protein and complement C3, representing a low inflammation profile, are causally associated with higher risk of AD in the general Danish population [45, 46]. However, the dynamics of IL-8 in the pathogenesis of neurological diseases needs further investigation.

### Strength and limitations

To the best of our knowledge, we firstly comprehensively tested the causal associations between multiple inflammatory markers with cognitive performance and measures of atrophy. By using two sample MR design, including the reverse MR study, we maximally avoided the drawbacks of reverse causation and residual confounding in observational studies. However, several limitations need to be taken into account when interpreting the results. First, the activities of the inflammatory markers are complex, particularly cytokines that are highly pleiotropic and can act on many different cell types [47, 48] and that depending on the context play roles in repair of tissue damage as well as in (chronic) inflammation. Moreover, individual cytokines can have many functions and different cytokines can share similar functions, which will induce a series of combined effects synergistically or antagonistically that functionally alter target cells [47, 48]. Despite a series attempts have been made by excluding variants that were identified for multiple cytokines and by performing sensitivity analyses to ensure the elimination of potential pleotropic effect, no established methodology deals with such a complex orchestrated traits’ network. Therefore, we could not completely rule out the possibility of bias by directional pleiotropy using current methods. In addition, given the fact that cytokines rarely manifest their effects alone but rather work in regulatory networks, gene–gene interaction is important to disentangle the role of inflammatory markers in health-related conditions [49], such as AD [50] and other forms of dementia. Second, we used a significance threshold of *p* < 5e − 6 for the selection of instrumental variables, and this might have included false-positive variants and consequently bias findings. However, most of the inflammatory markers are very expensive to measure; the included GWAS for these markers, although being the largest to date, comprises only 8293 European-ancestry individuals. Compared to the sample sizes of tens of thousands for other traits, for example, the outcome phenotypes, this might be too small to detect as many genome-wide significant genetic variants as possible. Therefore, the selection of a relaxed threshold this is a trade-off with statistical power (Fig. 2). A more stringent threshold results in less available instrumental variables (Table 1) with subsequently decreased statistical power. Consequently, a null association identified might not be indicative of absence of evidence, but rather of insufficient power. In our analyses, for some markers with available instruments at both thresholds, estimates for the same outcome are comparable (Supplementary Tables 1 and 3) but with smaller confidence interval using relaxed p-value threshold given increased power. Furthermore, in previous MR studies, when it comes to complex traits, such as depression, with limited genetic variants at *p* < 5e − 08 level, a more relaxed *p*-value (*p* < 1e − 6) has been adopted and successfully disentangled the bidirectional causal relationships between physical activity and depression in MR analyses [51]. Taken together, we also used a relaxed threshold to identify any possible link between systemic inflammatory markers with outcome. However, more studies are needed to confirm these possible associations, particularly using genetic variants from GWAS with larger sample size. Lastly, this study is performed based on populations of European ancestry thus the results could be not representative of other groups with different ethnic backgrounds.

## Conclusion

In conclusion, our MR study found some evidence to support a causal association of higher genetically determined IL-8 level and better cognitive performance and smaller hippocampal volume. Further research is needed to elucidate mechanisms underlying these associations, and to assess the suitability of these markers as potential preventive or therapeutic targets.

## Supplementary Information

Below is the link to the electronic supplementary material.

Supplementary file1 (XLSX 183 kb)
